# COVID-19 and radiation oncology: the experience of a two-phase plan within a single institution in central Italy

**DOI:** 10.1186/s13014-020-01670-9

**Published:** 2020-09-29

**Authors:** Luciana Caravatta, Consuelo Rosa, Maria Bernadette Di Sciascio, Andrea Tavella Scaringi, Angelo Di Pilla, Lucia Anna Ursini, Maria Taraborrelli, Annamaria Vinciguerra, Antonietta Augurio, Monica Di Tommaso, Marianna Trignani, Marianna Nuzzo, Maria Daniela Falco, Andrea De Nicola, Nico Adorante, Fabiola Patani, Giuseppe Centofanti, Lucrezia Gasparini, David Fasciolo, Fiorella Cristina Di Guglielmo, Cecilia Bonfiglio, Marzia Borgia, Gabriella Caravaggio, Stefano Marcucci, Consalvo Turchi, Domenico Mancinelli, Stephanie Sartori, Thomas Schael, Angelo Muraglia, Sergio Caputi, Claudio D’Amario, Nicoletta Verì, Domenico Genovesi

**Affiliations:** 1 Department of Radiation Oncology, “SS Annunziata” Hospital, “G. D’Annunzio” University, Via Dei Vestini, 66100 Chieti, Italy; 2 Department of Neuroscience, Imaging and Clinical Sciences, “G. D’Annunzio” University, Chieti, Italy; 3 Quality and Clinical Governance “SS Annunziata” Hospital, Chieti, Italy; 4 Management “SS Annunziata” Hospital, Chieti, Italy; 5 Rector “G. D’Annunzio” University, Chieti, Italy; 6 Abruzzo Region Health Policy Department, L’Aquila, Italy

**Keywords:** Coronavirus, COVID-19, Pandemic, Radiation oncology, Radiotherapy, SARS-CoV-2

## Abstract

**Background:**

COVID-19 in Italy has led to the need to reorganize hospital protocols with a significant risk of interruption to cancer treatment programs. In this report, we will focus on a management model covering the two phases of the COVID-19 emergency, namely lockdown-phase I and post-lockdown-phase II.

**Methods:**

The following steps were taken in the two phases: workload during visits and radiotherapy planning, use of dedicated routes, measures for triage areas, management of suspected and positive COVID-19 cases, personal protective equipment, hospital environments and intra-institutional meetings and tumor board management. Due to the guidelines set out by the Ministry of Health, oncological follow-up visits were interrupted during the lockdown-phase I; consequently, we set about contacting patients by telephone, with laboratory and instrumental exams being viewed via telematics. During the post-lockdown-phase II, the oncological follow-up clinic reopened, with two shifts operating daily.

**Results:**

By comparing our radiotherapy activity from March 9 to May 4 2019 with the same period in 2020 during full phase I of the COVID-19 emergency, similar results were achieved. First radiotherapy visits, Simulation Computed Tomography and Linear Accelerator treatments amounted to 123, 137 and 151 in 2019 compared with 121, 135 and 170 in 2020 respectively. There were no cases of COVID-19 positivity recorded either in patients or in healthcare professionals, who were all negative to the swab tests performed.

**Conclusion:**

During both phases of the COVID-19 emergency, the planned model used in our own experience guaranteed both continuity in radiotherapy treatments whilst neither reducing workload nor interrupting treatment and, as such, it ensured the safety of cancer patients, hospital environments and staff.

## Introduction

**Fig. 1:**
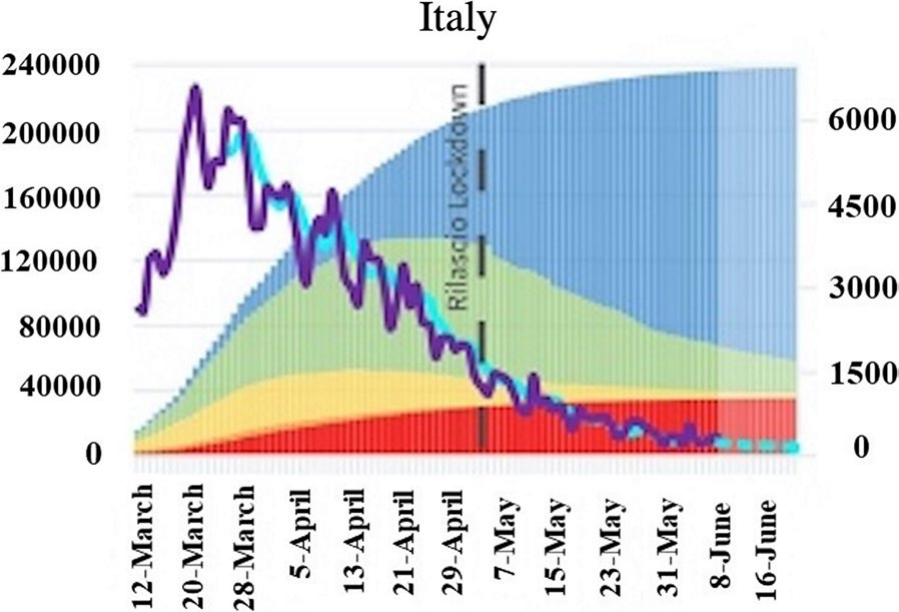
Trends and projections of Italy’s epidemic report. The purple line represents the number of new real daily cases (to the right of the reference scale); the light blue line represents the persistence conditions of the lockdown. In the background (to the left of the scale), the cumulative day-per-day deaths (red), recoveries (blue), patients currently positive and requiring admission to intensive units (orange), non-intensive unit admissions (yellow) and home self-isolation (green) (modified by https://ilsegnalatore.info/)

**Fig. 2:**
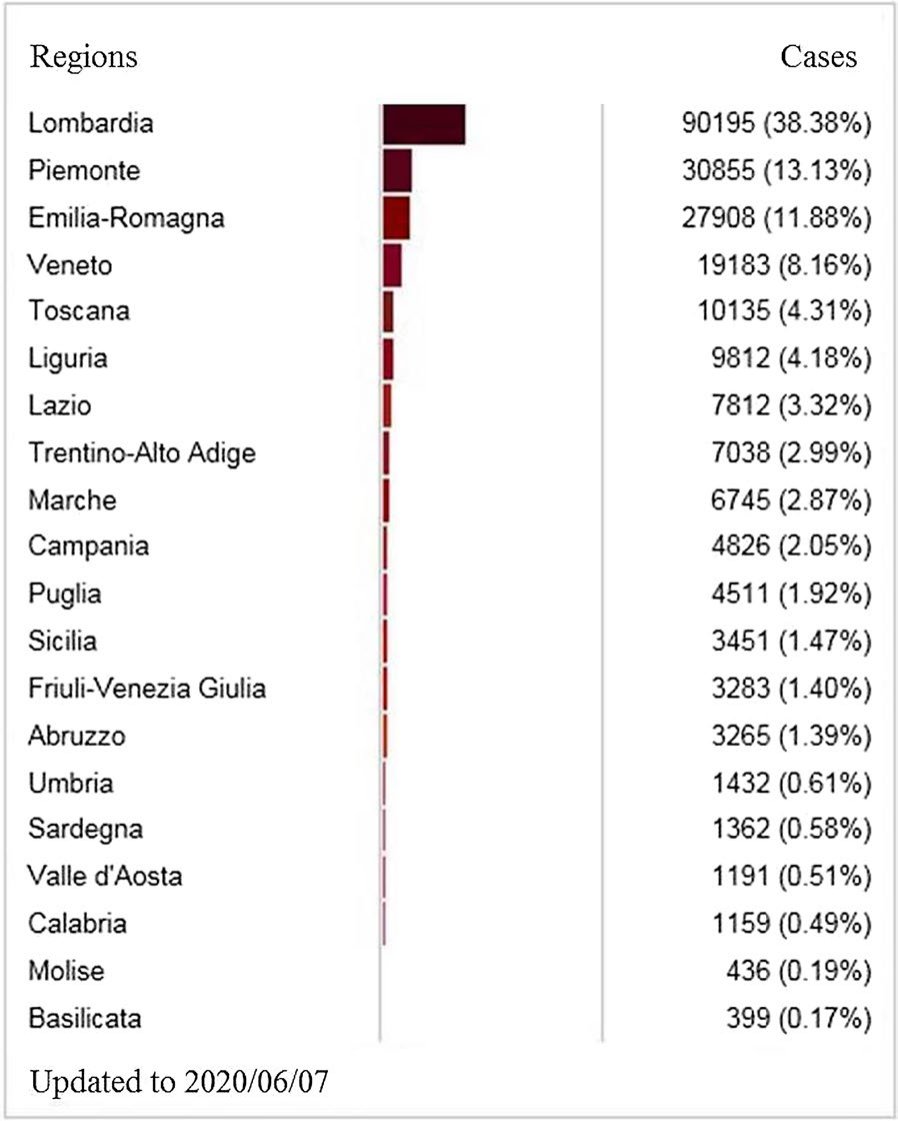
COVID-19 cases divided into individual Italian regions (modified by https://ilsegnalatore.info/)

The rapid and uncontrolled spread of COVID-19 in some regions and provinces of northern Italy led to the declaration of partial and complete lockdown of the entire country by the national government between March 8 and March 22, 2020 and this phase, known here as Phase I, lasted from March 9 to May 3, 2020 [1–3]. From May 4, 2020 the so-called Phase II was approved, and consisted in a gradual deceleration to Phase I as a result of a decline to the epimedic's curve [4] (Fig. 1). On June 7, 2020 Italy recorded a total of 234,998 COVID-19 cases wherein: 168,640 had recovered, 32,459 were in home self-isolation and 33,899 deceased [5]. Until the middle of April, the number of patients hospitalized in intensive and non-intensive care units progressively increased as a result of the consequent need for a complete reorganization of hospital infrastructures. The territorial heterogeneity of the COVID-19 trend brought about a decisive and dramatic prevalence in areas of northern Italy and a more contained incidence in areas of central and southern Italy (Fig. 2) [5].

**Fig. 3:**
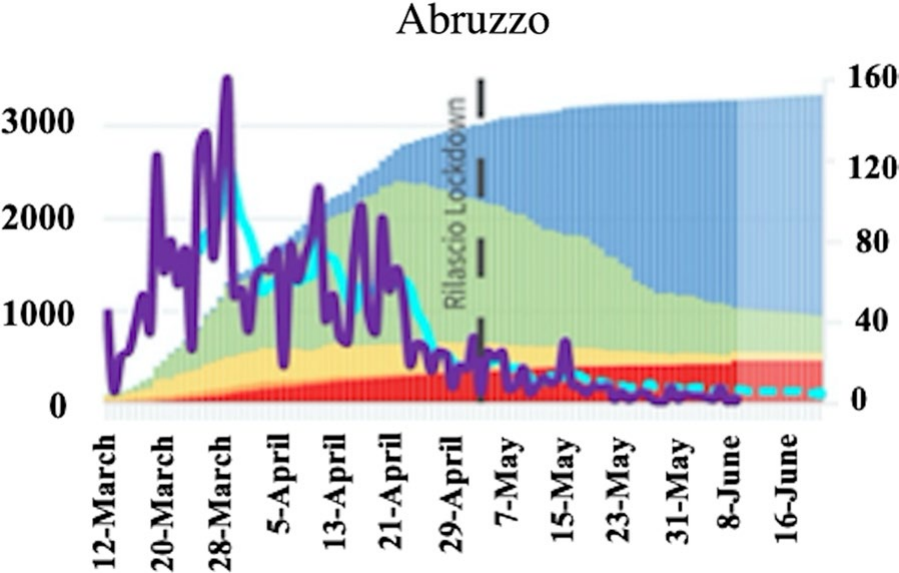
Trends and projections of the Abruzzo epidemic report. The purple line represents the number of new real daily cases (to the right of the reference scale); the light blue line represents the persistence conditions of the lockdown. In the background (scale on the left), the cumulative day-per-day deaths (red) and recoveries (blue), patients currently positive and requiring admission to intensive units (orange), non-intensive unit admissions (yellow) and home self-isolation (green) (modified by https://ilsegnalatore.info/)

In Abruzzo, a region of central Italy with a population of 1,304,970 inhabitants, a total number of 3265 (1.39%) cases were recorded on June 7, 2020 with a spike in infections taking place on March 29, 2020, and resulted in hospitals operating at maximum capacity on April 3, 2020, with 418 deaths, 2215 recoveries and 632 in home self-isolation [6] (Fig. 3). Therefore, although with smaller numbers, the Abruzzo region also underwent an important distress and COVID-19 centered reorganization of hospital activities with the interruption on March 13, 2020 of all planned medical and surgical activities with the exception of oncology and hematology services which clinicians judged non-deferrable. In fact, when regarding cytostatic, radiation oncology or immunological treatments, the Ministry of Health emphasized to local Health Authorities the need to guarantee these "life-saving" treatments to all patients and to plan dedicated routes and spaces for those patients being treated as well as the availability of all necessary individual protective devices and equipment [7, 8]. Furthermore, the Italian Association of Radiotherapy and Clinical Oncology (AIRO) implemented a document to make radiotherapy operating procedures homogeneous throughout the ongoing COVID-19 emergency [9] and conducted a targeted national survey [10]. To this regard, several experiences have been published on the management of radiation therapy during the pandemic [11–17]. We aim to report here our particular experience in the organizational planning of radiotherapy during the two phases of the COVID-19 emergency, lockdown phase I and post-lockdown phase II. It was in doing so that we were able to guarantee treatment continuity whilst maintaining the safety of cancer patients and healthcare personnel within the framework of a complete COVID-19 reorganization at the University Hospital of Chieti.

## Materials and methods

The Radiation Oncology Center of Chieti is one of four radiotherapy centers in the Abruzzo region and is equipped with two Linear Accelerators (Linacs) that both operate during two daily shift patterns between 8.00 am and 8.30 pm; Simulation Computed Tomography (Simul CT) and clinical dosimetry facilities that work at normal capacity within the working hours of 8.00 am and 2.00 pm; a clinic room dedicated to first radiotherapy visits and a clinic room dedicated to the scheduled follow-up visit of cancer patients both with working hours between 8.00 am and 2.00 pm; and finally an oncological day-hospital unit for the management of concurrent radio-chemotherapy treatments.

The radiotherapy staff consist of 11 doctors, 14 technicians, 3 dedicated physicists, 5 nurses, 2 administrative staff members, a socio-health operator, and a secretary. The center is also a university site with a specialization school in Radiation Oncology and a total of 7 resident doctors and a staff operating across 44 units. An average of around 800 patients are treated each year. Table 1 shows the measures planned for radiotherapy activities between the Hospital Management and Hospital Quality Office respectively in both lockdown phase I and post-lockdown phase II of the COVID-19 pandemic.

**Table 1:** Planned actions implemented for radiotherapy activities in lockdown Phase I and in post-lockdown Phase II in the experience of Chieti Radiation Oncology department

Nos.	Planned actions	
	Phase I: Lockdown	Phase II: Post-lockdown
1	Full maintenance of Radiotherapy treatments on both Linacs	As per Phase I
2	Linacs disinfection at each workshift	As per Phase I
3	Preference for hypofractionated schemes	As per Phase I
4	Full maintenance of Simul CT and Dosimetry activities	As per Phase I
5	Simul CT disinfection at each workshift	As per Phase I
6	Staff: systematic hand washing before and after each clinical and technical procedure	As per Phase I
7	Maintenance of a single clinic room for the first radiotherapy visits. Interruption of oncological follow-up clinic room with phone contact of patients and viewing of laboratory and instrumental exams via telematics. On urgency, patients are booked in the single clinic room active	Full recovery of the oncological follow-up clinic room clinic with double daily shift 8.00 am–1.00 pm and 2.00 pm–5.00 pm with spacing appointments of 1 patient every 45 min
8	Preparation of 2 dedicated areas outside the waiting rooms for family members and carers. Entry into the Radiation Oncology center reserved for one family member and only for the first radiotherapy visits or on urgent cases	Preparation of a single pre-waiting room area for family members and carers. Entry into the Radiation Oncology center reserved for one family member and only for the first radiotherapy visits or on urgent cases
9	Triage area with nursing staff: (a) entry for 4 patients at a time with a distance of at least 1 m; (b) body temperature detection with Thermo can; (c) finding of respiratory symptoms, ocular disorders (conjunctivitis), dysgeusia and anosmia; (d) contacts with suspected COVID-19 by filling of the dedicated Hospital questionnaire; (e) obligation of surgical mask for patients and carers [18–21]	As per Phase I
10	Management of suspected case in triage for patients, staff, carers and third parties: if temperature ≥ 37.5° repetition after 10 min and if confirmed, access to the center is not allowed. Evaluation for deferral of planned clinical or technical performance: in the case of deferral, the patient is rescheduled; in the case of non-deferral, the patient accesses the service by adopting all the safety criteria indicated in points 12 and 13 [19]	As per Phase I
11	Double daily shift of all staff in order to prevent potential multiple infections	**/**
12	Personal protective equipment. (a) Visits: surgical mask and gloves; FFP2 mask with superimposed surgical mask in patients with respiratory symptoms; (b) Simul TC and Linear Accelerators: FFP2 mask with superimposed surgical mask and single-use gloves; systematic hand disinfection; visor or protective glasses for Head and Neck and respiratory tumors and for patients with respiratory symptoms [18–21]	As per Phase I
13	Symptomatic and asymptomatic positive COVID-19 patient: medical evaluation for treatment interruption based on the clinical disease status, with monitoring of the clinical status and treatment recovery after 2 consecutive negative swabs, symptomatic absence and negative CT scan. In the case of treatment continuation because it cannot be deferred: preparation of separate paths; bunker disinfection before and after treatment; FFP2 masks with superimposed surgical mask; single-use gloves and gowns; visors or protective glasses and overshoes for staff; separate and disinfected room for dressing and undressing [21]	As per Phase I
14	Maintenance of Department meetings for discussion of clinical cases and ongoing scientific work with limited number of professionals and spacing measures	Full recovery of Department Meetings without contingent number of professionals but with maintenance of the safety distance of at least 1 m
15	Maintaining of multidisciplinary Tumor Board meetings only by requesting consultations, e-mail correspondence, phone contacts and telematic platforms	Full recovery of multidisciplinary Tumor Board meetings

## Results

By comparing the center’s treatment activity under ordinary routine conditions, within the period March 9–May 4, 2019, with the same time interval during full lockdown phase I of the COVID-19 emergency, March 9–May 4, 2020, no changes were observed in the number of services performed. Significantly, in 2019, 123 first radiotherapy visits were performed, with 137 new patients prepared for Simul CT and 151 patients treated on Linear Accelerators. In the same period in 2020 and during the critical phase of the pandemic, the action plans set up allowed for 121 first radiotherapy visits to be performed, as well as 135 new patients to be prepared for Simul CT and 170 patients to be treated on Linear Accelerators (Table 2). Dose hypofractionation was predominantly used in breast, prostate and palliative treatment. There were no cases of COVID-19 positivity recorded in patients or in healthcare professionals throughout both phase I and phase II. During the week of May 25 to May 31, 2020 all staff members of the Center underwent oropharyngeal and nasopharyngeal swab testing with negative results in all 42 examined. Two staff members have not been examined, due to their being in maternity and illness periods respectively since last year.

**Table 2:** Comparison of the number of radiotherapy performances in the lockdown Phase I (March 9–May4, 2020) with the same period of 2019 in ordinary clinical activity

	Time: March 9–May 4, 2019	Time: March 9–May, 4, 2020
First radiotherapy visit	123	121
New patients prepared for Simul CT	137	135
Patients treated on LINACS	151	170

## Discussion

The COVID-19 pandemic that hit Italy had serious repercussions on the national hospital system which subsequently had to reorganize its assistance network with the introduction of COVID-19 centered hospitals and with a prevalence of intensive and sub-intensive care units. Many internal and surgical departments were reconverted and dedicated to the management of COVID-19 within only a few weeks, with the radiological and laboratory diagnostic activities seriously involved in these plans. Although witnessing more contained case numbers than the regions and provinces of northern Italy, the main hospitals of the Abruzzo region and, specifically in our experience, the University Hospital of Chieti, were reorganized to conform to COVID-19 measures in only a short period of time. This particular situation carried a double risk due to the potential of delays or interruptions in to oncological therapies, which, despite being a "life-saving" treatment, could have been hazardous to patients who are often already frail and also at high risk of infection themselves as well as to carers and health personnel alike. Several scientific documents and papers published on the subject represented an important reference point especially in the first phase of the emergency [11–17] and with particular regard to the use of protection systems, triage procedures and the use of hypofractionation schemes. In particular, a national survey conducted by the Italian Society of Radiotherapy and Clinical Oncology and, prior even to this, a survey of the radiotherapy centers of the Lombardy region, the region most affected by COVID-19 [10, 14], both reported and supported the measures, implemented for the new rearrangement of centers, the workloads necessary and the COVID-19 cases found. Most of the measures adopted and reported in the surveys are in line with the planned approach undertaken in our particular experience with the exception of the "working from home scheme" which cannot be performed at our center due to the absence of dedicated technology.

During both lockdown and post-lockdown phases, we performed a full maintenance of simul-CT, dosimetry activities and radiotherapy treatment, preferring the incorporation of hypofractionated schemes. Both Linac and simul-CT disinfection was guaranteed during each shift as well as strict hand hygiene with the use of hydroalcoholic solutions and disposable gloves mandatory to all staff, both before and after each clinical and technical procedure.

For personnel and patients alike, temperature (required < 37.5 °C) was checked prior to entering the Department, as well as the mandatory wearing of surgical masks. In the triage area, only four patients at a time were permitted entry, with a distance of at least one meter. Respiratory symptoms, dysgeusia and anosmia and ocular disorders, such as conjunctivitis, were also checked; whilst furthermore, a dedicated hospital questionnaire on any potential contact with suspected COVID-19 patients was issued and completed by all patients.

In accordance with the Ministry of Health guidelines, oncological follow-up visits were interrupted during the lockdown-phase I: consultation by telephone was made with patients, with laboratory and instrumental exams viewed via telematics. During phase II, the oncological follow-up clinic reopened, with two shifts daily (8.00 am–1.00 pm and 2.00 pm–5.00 pm), and appointment spacing of one patient every 45 min.

Overall, the clinical activity of the Italian centers saw a reduction of < 10% in 32% of cases and of 10–30% in 30.4% of cases. In our particular experience, the clinical activity during the lockdown phase was comparable to that of the same period of 2019 and, except for a rescheduling of the oncological follow-up visits, there were no interruptions in treating patients nor reduction in radiotherapy preparation or treatment of newly diagnosed cancer patients. The national survey also reports 29.6% of the positive or suspect cases treated in Italian centers. No positive or suspicious cases were found in our experience during phase I and phase II. However, the differences shown in the reduction of workloads and the number of positive or suspect patients reported in the national survey highlight the predominance of the COVID-19 cases in the regions of northern Italy and, in particular, Lombardy where a reduction of 10–50% in radiotherapy was registered, but also in greater organizational awareness due to the increase of national and international guidelines emerging on the subject during this period and in the detailed recommendations on individual tumor pathologies treated using hypofractionated regimens [22–26]. In our experience, dose hypofractionation has been used in breast, prostate and palliation cancers.

## Conclusion

The organizational model implemented in our experience, respectively, during the lockdown phase I and the post-lockdown phase II ensured an optimal continuity of radiotherapy workflow without reducing workload or interrupting the radiation therapy cycles, as well as the safety of cancer patients, the hospital environments and the radiation oncology staff. It is therefore recommended that each Radiation Oncology center customize its organizational model for the management of COVID-19 based on the characteristics and directives used in the University Hospital of Chieti and on the specific features of the center in terms of equipment, staff and hospital environments.

## Data Availability

Not applicable.

## Abbreviations

AIRO: Italian association of radiotherapy and clinical oncology
Linacs: Linear accelerators
Simul CT: Simulation computed tomography
